# Correlation of Alberta Stroke Program Early Computed Tomography (CT) Score Among Ischaemic Stroke Patients and Their Outcomes at Selected Centres in Kampala: A Prospective Study

**DOI:** 10.7759/cureus.86729

**Published:** 2025-06-25

**Authors:** Judith Mutesi, Faith Ameda, Robert Mukisa, Praise Akatukunda, Rita Nassanga

**Affiliations:** 1 Radiology, Makerere University, College of Health Sciences, Kampala, UGA; 2 Neurology, Mulago National Referral Hospital, Kampala, UGA; 3 Statistics, Makerere University Joint AIDS Program, Kampala, UGA

**Keywords:** aspects, functional outcomes, ischemic stroke, modified rankin scale, neuroimaging

## Abstract

Background

Stroke contributes significantly to morbidity and is the second leading cause of death globally. The Alberta Stroke Program Early Computed Tomography (ASPECT) Score, a 10-point scoring system with anatomical regions of the brain over the middle cerebral artery (MCA) territory, is used to assess early ischaemic changes on a non-contrast computed tomography scan (NCCT) of the head. It is used as a predictor of functional outcome and in the stratification of ischaemic stroke patients’ treatment to guide reperfusion and non-thrombolytic management. This study aimed to determine the correlation between the ASPECTS and the outcomes of ischaemic stroke patients in Kampala, Uganda.

Methods

This was a prospective study carried out at three selected centres. All patients diagnosed with ischaemic stroke involving the MCA underwent NCCT of the head to determine the ASPECTS. Enrolled participants were index admissions to the hospital following the symptoms and signs of stroke. Patient demographics, clinical presenting complaints, Glasgow Coma Scale (GCS), comorbidities, and medical and treatment history for individual patients were documented in the data collection tool. Study patients were recruited consecutively and followed up one month later using the Modified Rankin Scale (mRS). The participants’ outcomes were described as favourable outcomes and poor outcomes. Spearman correlation analysis was carried out to measure the strength and direction of association between ASPECTS and mRS.

Results

The average ASPECTS score was 5.71, SD ± 3.4, with a left hemisphere predilection. There was a significant inverse correlation between ASPECTS and mRS scores (r = -0.618, p < 0.001). 17% (n=20) had favourable outcomes (mRS, 0-2), whereas 83.1% (n=98) had poor outcomes (mRS, ≥3) with 26.27% mortality. Poor outcomes among patients were associated with overall lower ASPECTS, no education, low GCS at admission, involvement of M3 and M5 brain regions and additional vascular territory-posterior cerebral artery involvement.

Conclusion

The inverse correlation between the ASPECTS and mRS seen in this study highlights the value of the ASPECTS as a reliable tool to predict functional outcomes in ischaemic stroke patients. The poor outcomes observed reflect the need to utilise the ASPECTS to ensure appropriate timely interventions and rehabilitation and for individualised stroke protocols in our hospitals to initiate thrombolytic and thrombectomy therapy.

## Introduction

Stroke is the second leading cause of death globally and a major cause of disability [1]. Globally, ischaemic strokes account for 62% of all incident strokes, whereas haemorrhagic strokes account for 28% of all incident strokes [2].

There are various modalities for neuroimaging which include magnetic resonance imaging (MRI), computed tomography (CT) scan, and computed tomography angiography (CTA) [3]. A head CT scan is the preferred choice of neuroimaging because it is fast, relatively available and cost-effective [4]. Following thrombolytic therapy for ischaemic stroke, brain herniation and intracranial haematoma may occur; head NCCT is used to screen for these possible complications [5,6]. The Alberta Stroke Program Early Computed Tomography (ASPECT) Score is a 10-point scoring system with anatomical regions of the brain over the middle cerebral artery (MCA) territory used to assess early ischaemic changes. The score was developed for standardised lesion assessment on NCCT as a predictor for functional outcome and the symptomatic intracranial haemorrhage after intravenous thrombolysis among patients with a score of less than or equal to seven [7]. The ASPECTS has been applied in the African context in research studies to determine its correlation with clinical scores and functional outcomes [8]. 

In Uganda, ischaemic stroke is one of the leading causes of morbidity, disability and mortality. A study done in Uganda on stroke mortality outcomes showed a 30-day mortality of 26.8% [9]. The financial burden of stroke is evidenced by the increased hospitalisations, prolonged hospital stays, the required rehabilitation of stroke patients' permanent disability and consequent inability to earn. In Uganda, the ASPECTS score utilisation in our setting is very low or non-existent based on the current studies that have been done [10]. The delay in accessibility of the NCCT and under-utilisation of the ASPECTS greatly limits the diagnosis of acute stroke and timely referral of patients for thrombolysis. Utilisation of the ASPECTS in the management of these ischaemic stroke patients will improve post-stroke function and prevent mortality and stroke-associated morbidity for those patients who can benefit from thrombolytic therapy. This study sought to describe the correlation between the ASPECTS among ischaemic stroke patients and their outcomes at three selected centres in Kampala, Uganda.

## Materials and methods

Study design and sample recruitment

This was a prospective observational study conducted over a period of five months (Jan 2024-May 2024). The study was conducted at Mulago National Referral Hospital, Kiruddu National Referral Hospital and St. Francis Nsambya Hospital. Ethics approval to carry out the study was obtained from the Department of Radiology and Radiotherapy, Makerere University - School of Medicine Research and Ethics Committee (SOMREC) under Mak-SOMREC-2023-741, whereas administrative clearance was obtained from Mulago and Kiruddu National Referral Hospitals and St Francis Hospital Nsambya Research Ethics Committees. Informed consent was obtained from all participants. The study population were all patients with ischaemic stroke confirmed on CT scan involving the middle cerebral artery at Mulago and Kiruddu National Referral Hospitals and St Francis Hospital Nsambya. Our inclusion criteria were all adult patients aged 18 years and above with an index clinical diagnosis of stroke during the study period that was confirmed as an ischaemic stroke on a CT scan. Our exclusion criteria were patients with a previous history of stroke, isolated posterior circulation stroke and isolated anterior cerebral artery (ACA) stroke. Patients with an initial diagnosis of ischaemic stroke confirmed on CT scan imaging and who had given informed consent were enrolled on the study. Consecutive sampling was done to recruit participants until the sample size was realised. For this study, the sample size calculation was based on the sample size estimation charts for Spearman correlation. A study done in Egypt that showed the correlation between the ASPECT score and the functional outcomes based on the modified Rankin scale assessed at three months showed a negative correlation with a coefficient r = 0.39 [8]. Based on the assumption that patients in our setting present much later after symptom onset and at one month of assessment have much poorer outcomes, our coefficient r was set at 0.6, and the sample size was estimated to be 120.

Methodology

Patient demographics, clinical presenting complaints, Glasgow Coma Scale (GCS), comorbidities, and medical and treatment history for individual patients were documented in the data collection tool (Table 5 in Appendices). The CT scan images were later evaluated by the principal investigator in consultation with a general radiologist to determine the ASPECTS for these participants, and this was documented in the data collection tool (Table 6 in the Appendices). At one-month post entry into the study, phone calls were made to the participants and their next of kin to determine the functional outcomes using the Modified Rankin Scale and any rehabilitation services received. This data was documented in the data collection tool (Table 7 in Appendices).

Statistical analysis

Data entry was made using the EpiData version 3.0 software package. The final data was exported to STATA version 14.0 for cleaning and analysis. Descriptive analysis was conducted to describe continuous variables expressed as frequencies and percentages (%). Mean, standard (±SD) and median interquartile range [IQR] were used for continuous variables which were normally distributed and skewed, respectively. Spearman correlation analysis was carried out to measure the strength and direction of association between ASPECTS, mRS, MCA individual regions and age of patients. The participants’ outcomes were described as favourable outcomes and poor outcomes. Favourable outcomes were determined by having a Modified Rankin Scale (mRS) between 0 and 2, whereas poor outcomes were described as having an mRS ≥ 3. The Pearson chi-square was used as a test to identify the relationship between patient characteristics and their outcomes at bivariate analysis. To determine the independent variables associated with outcomes, an arbitrary P-value of 0.25 was used, and all variables with a P-value greater than 0.25 were excluded from the multivariable analysis. Adjusted odds ratios from a multivariable binary logistic regression model were used to determine the variables statistically associated with outcomes among ischaemic stroke patients. The LR chi² was used to determine 5% significance, and the overall significance of the model was < 0.001.

## Results

Of the 120 patients enrolled in the study, two of them were lost to follow-up, and 118 were followed up successfully. Their demographic characteristics are summarised in Table 1.

**Table 1:** Demographic characteristics of ischaemic stroke patients at MNRH, KNRH and SFN N = 120 MNRH: Mulago National Referral Hospital, KNRH: Kiruddu National Referral Hospital, SFN N: St. Francis Hospital Nsambya Descriptive analysis was conducted to describe continuous variables expressed as frequencies and percentages (%). Mean, standard (±SD) and median interquartile range (IQR) were used for continuous variables which were normally distributed and skewed, respectively.

Characteristics of respondents	Frequency	Proportion	95% CI
Age (years)	Median, 60	IQR (48 - 72)	
Sex			
Female	68	56.67	0.47 – 0.66
Male	52	43.33	0.34 – 0.52
Age group (years)			
≤49	36	30.00	0.22 – 0.39
50 – 59	18	15.00	0.09 - 0.23
60 – 69	34	28.33	0.20 – 0.37
≥70	32	26.67	0.19 – 0.36
Nationality			
Non-Ugandan	6	5.00	0.19 – 0.11
Ugandan	114	95.00	0.89 – 0.98
Urbanity			
Rural	50	41.67	0.33 – 0.51
Urban	70	58.33	0.49 – 0.67
Education Level			
None	29	24.17	0.17 – 0.33
Primary	22	18.33	0.11 – 0.26
Secondary	41	34.17	0.26 – 0.433
Tertiary	28	23.33	0.14 – 0.30

On average, the ASPECTS was 5.71, SD ± 3.4, range: (0-10); the scores were identified from both cerebral hemispheres independently depending on which was affected. There were higher scores identified in the left cerebral hemisphere compared to the right cerebral hemisphere (n=70 vs. n=61). Overall, the left had a mean ASPECTS of 5.68, SD ± 3.3, whereas the right had a mean ASPECTS of 5.95, SD ± 3.4. Among the high scores, score 9 had the highest frequency of 24 (20%), and among the low scores, ASPECTS-0 had a high frequency of 12 (10%). Some patients had other concurrent vascular territories involved, and these were the posterior cerebral artery (PCA) (n=22, 18%) and anterior cerebral artery (ACA) (n=3, 3%). The functional outcomes at one month were assessed using the Modified Rankin Scale with scores between 0 and 6; there were two patients with undocumented mRS scores because they were lost to follow-up. The disaggregation of mRS scores is shown in Table 2.

**Table 2:** mRS scores of ischaemic patients admitted at MNRH, KNRH and SFN (N=118) MNRH: Mulago National Referral Hospital, KNRH: Kiruddu National Referral Hospital, SFN N: St. Francis Hospital Nsambya Functional outcomes were determined by the Modified Rankin Scale (mRS); favourable outcomes were described as between 0 and 2, whereas poor outcomes were described as having mRS ≥3. The mRS scores were expressed as frequency and proportion.

mRS scores	Frequency	Proportion
0	1	0.85
1	5	4.24
2	14	11.86
3	18	15.25
4	33	28.00
5	16	13.56
6	31	26.27

**Figure 1:**
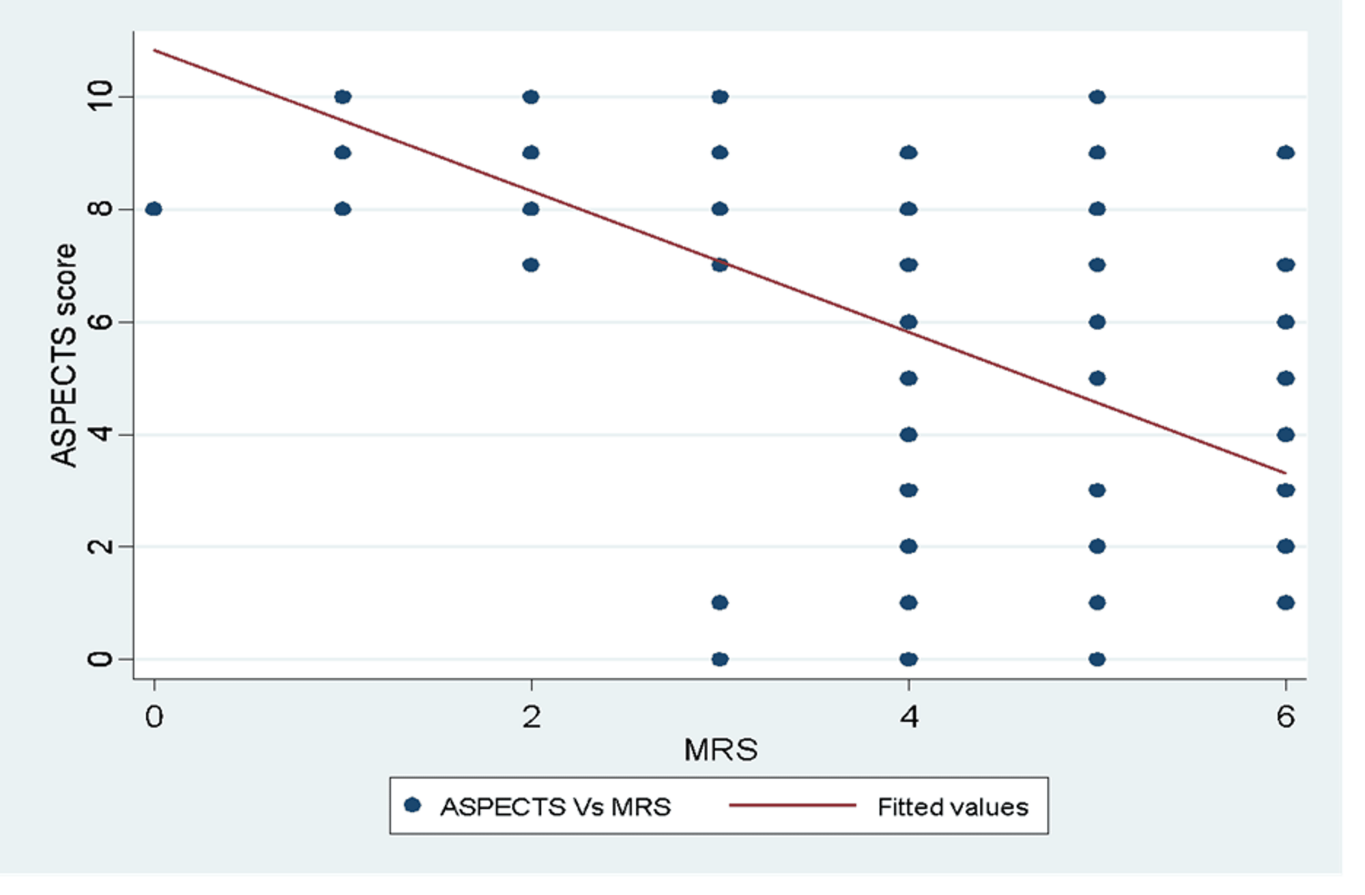
A scatter plot illustrating a high inverse correlation between ASPECT scores and mRS scores among patients diagnosed with ischaemia at MNRH, KNRH and SFN MNRH: Mulago National Referral Hospital, KNRH: Kiruddu National Referral Hospital, ASPECT: Alberta Stroke Program Early Computed Tomography, mRS: Modified Rankin Scale Spearman correlation analysis was carried out to measure the strength and direction of association between ASPECTS and mRS. There was a significantly high inverse correlation between ASPECT scores and mRS scores (r = -0.618, p < 0.001).

There was a significantly high inverse correlation between ASPECTS and mRS scores (r = -0.618, p< 0.001). A unit increase in ASPECTS yields a high decrease in mRS scores (Figure 1).

**Figure 2:**
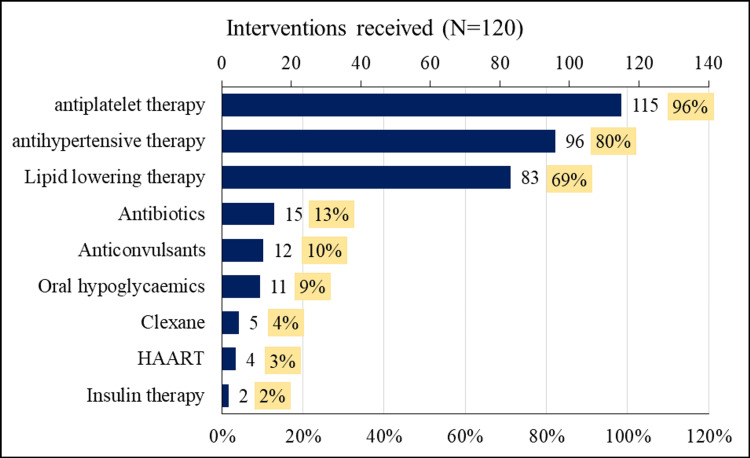
Distribution of interventions received by ischaemic stroke patients at MNRH, KNRH and SFN MNRH: Mulago National Referral Hospital, KNRH: Kiruddu National Referral Hospital, ASPECT: Alberta Stroke Program Early Computed Tomography, mRS: Modified Rankin Scale, SFN: St. Francis Hospital Nsambya The interventions received were expressed as frequency and percentages (%).

Variables which had a significantly very high inverse correlation with ASPECTS were M2 (MCA cortex lateral to insular ribbon) (r = -0.845, p< 0.001), M3 (posterior MCA) (r = -0.756, p < 0.001) and M5 (lateral cortex immediately rostral to M2) (r = -0.753, p < 0.001). It was also realised that M6 (posterior cortex immediately rostral to M3) and M4 (anterior cortex immediately rostral to M1) also had a significantly high inverse correlation with ASPECTS (r = -0.656, p < 0.001) and (r = -0.688, p < 0.001), respectively. However, a significant medium inverse correlation was realised between Caudate and ASPECTS, respectively (r = -0.426, p < 0.001). Patients received interventions ranging from antiplatelet therapy (n=115, 96%) and antihypertensive therapy (n=96, 80%) to insulin therapy (n=2, 2%). The distribution of interventions received is in Figure 2.

Among other intervention services received were rehabilitation services. A total of 37 (31%) patients received rehabilitation services, which included mostly physiotherapy; two patients received more than one rehabilitation service, i.e., speech and language therapy and occupational therapy.

A total of 17% (n=20) had favourable outcomes (mRS, 0-2), whereas 83% (n=98) had poor outcomes (mRS, ≥3). Among patients who had poor outcomes, mortality was 26.27% (31/118). A chi-square test was conducted at bivariate analysis to determine the relationship between favourable and poor outcomes, and the statistically significant factors were education level and GCS ≤13. There were significantly poorer outcomes among patients with no education and a secondary level of education, comprising 27% and 36%, respectively (p= 0.019). Regarding GCS levels at admission, poor outcomes were observed at GCS levels (3-8 and 9-13) (n= 9 and 51, p= 0.010), respectively. Pertaining to presenting symptoms, there were poorer outcomes among patients with aphasia (n=49, p= 0.001) whereas in terms of other territories involved, there was a significantly high number of patients with poor outcomes among those with PCA involvement (n=21, p= 0.086). Patients with a tertiary education level (n=10, p= 0.019), dysarthria as a presenting symptom (n=9, p=0.063) and those who received rehabilitative physiotherapy (n= 12, p= 0.002) had favourable outcomes as evidenced by the p-values as shown in Table 3.

**Table 3:** Bivariate analysis to determine the relationship among outcomes of patients GCS: Glasgow Coma Scale, PCA: Posterior Cerebral Artery, MCA: Middle Cerebral Artery, ACA: Anterior Cerebral Artery, M1: First Segment of the Middle Cerebral Artery, M2: Second Segment of the Middle Cerebral Artery, M3: Third Segment of the Middle Cerebral Artery, M4: Fourth Segment of the Middle Cerebral Artery, M5: Fifth Segment of the Middle Cerebral Artery, M6: Sixth Segment of the Middle Cerebral Artery In bivariate analysis, the Pearson chi-square was used to test to identify the relationship between patient characteristics and their outcomes. To determine the independent variables associated with outcomes, an arbitrary p-value of 0.25 was used.

Variable (N=118)	Favourable outcomes n (%)	Poor outcomes n (%)	Total N (%)	Chi-square value (χ^2^)	P-Value
Sex					
Female	9 (45.00)	58 (59.18)	67 (56.78)	1.3617	0.243
Male	11 (55.00)	40 (40.82)	51 (43.22)		
Age group (years)					
≤49	9 (45.00)	26 (26.53)	35 (29.66)	3.3418	0.342
50 - 59	3 (15.00)	14 (14.29)	17 (14.41)		
60 - 69	5 (25.00)	29 (29.59)	34 (28.81)		
≥70	3 (15.00)	29 (29.59)	32 (27.12)		
Nationality					
Non-Ugandan	1 (5.00)	5 (5.10)	6 (5.08)	0.0004	0.985
Ugandan	19 (95.00)	93 (94.90)	112 (94.92)		
Urbanity					
Rural	10 (50.00)	38 (38.78)	48 (40.68)	0.8672	0.352
Urban	10 (50.00)	60 (61.22)	70 (59.32)		
Education level					
None	2 (10.00)	26 (26.53)	28 (23.73)	9.8958	0.019
Primary	2 (10.00)	19 (19.39)	21 (17.80)		
Secondary	6 (30.00)	35 (35.71)	41 (34.75)		
Tertiary	10 (50.00)	18 (18.37)	28 (23.73)		
GCS					
3-8	0 (0.00)	9 (9.18)	9 (7.63)	9.2487	0.010
9-13	5 (25.00)	51 (52.04)	56 (47.46		
14-15	15 (75.00)	38 (38.78)	53 (44.92)		
Comorbidities					
Cardiovascular disease	3 (15.00)	15 (15.31)	18 (15.25)	0.0012	0.972
HIV	1 (5.00)	9 (9.18)	10 (8.47)	0.3748	0.540
Renal disease	1 (5.00)	2 (2.04)	3 (2.54)	0.5870	0.444
Diabetes mellitus	4 (20.00)	16 (16.33)	20 (16.95)	0.1592	0.690
Hypertension	16 (80.00)	81 (82.65)	97 (82.20)	0.0799	0.777
Presenting symptoms					
Facial paralysis	7 (35.00)	17 (17.35)	24 (20.34)	3.1948	0.074
Hemiparesis	20 (100.00)	92 (93.88)	112 (94.92)	1.2901	0.256
Sensory loss of face	2 (10.00)	7 (7.14)	9 (7.63)	0.1925	0.661
Aphasia	2 (10.00)	49 (50.00)	51 (43.22)	10.8296	0.001
Dysarthria	9 (45.00)	24 (24.49)	33 (27.97)	3.4685	0.063
Other vascular territory					
PCA	1 (5.00)	21 (21.43)	22 (18.64)	2.9560	0.086
ACA	0 (0.00)	3 (3.06)	3 (2.54)	0.6282	0.428
Rehabilitation services					
Physiotherapy	12 (60.00)	25 (25.51)	37 (31.36)	9.1801	0.002
Occupational therapy	0 (0.00)	1 (1.02)	1 (0.85)	0.2066	0.650
Speech and language therapy	0 (0.00)	2 (2.04)	2 (1.69)	0.415	0.519
Other interventions					
Clexane	0 (0.00)	5 (5.10)	5 (4.24)	1.0656	0.302
HIV antiretroviral therapy	0 (0.00)	4 (4.08)	4 (3.39)	0.8450	0.358
Antibiotics	0 (0.00)	15 (15.31)	15 (12.71)	3.5070	0.061
Anticonvulsants	0 (0.00)	12 (12.24)	12 (10.17)	2.7262	0.099
Oral hypoglycaemic	2 (10.00)	9 (9.18)	11 (9.32)	0.0131	0.909
Insulin therapy	0 (0.00)	2 (2.04)	2 (1.69)	0.4152	0.519
Lipid lowering therapy	15 (75.00)	68 (69.39)	83 (70.34)	0.2508	0.617
Antiplatelet therapy	20 (100.00)	93 (94.90)	113 (95.76)	1.0656	0.302
Antihypertensive therapy	16 (80.00)	78 (79.59)	94 (79.66)	0.0017	0.967
MCA regions - abnormal					
Caudate (U)	4 (20.00)	36 (36.73)	40 (33.90)	2.0760	0.150
Internal Capsule (IC)	3 (15.00)	40 (40.82)	43 (36.44)	4.7797	0.029
Lentiform nucleus (IL)	4 (20.00)	52 (53.06)	56 (47.46)	7.2811	0.007
Insular Ribbon (IR)	3 (15.00)	58 (59.18)	61 (51.69)	12.9855	<0.001
M1	2 (10.00)	47 (47.96)	49 (41.53)	9.8566	0.002
M2	2 (10.00)	55 (56.12)	57 (48.31)	14.1501	<0.001
M3	0 (0.00)	43 (43.88)	43 (36.44)	13.8068	<0.001
M4	1 (5.00)	50 (51.02)	51 (43.22)	14.3349	<0.001
M5	2 (10.00)	56 (57.14)	58 (49.15)	14.7703	<0.001
M6	3 (15.00)	44 (44.90)	47 (39.83)	6.1953	0.013
Both cerebral hemispheres	1 (5.00)	8 (8.16)	98 (83.05)	0.2359	0.627
Individual hemisphere (N=109)					
Left hemisphere	9 (47.37)	51 (56.67)	60 (55.05)	0.5481	0.459
Right hemisphere	10 (52.63)	39 (43.33)	49 (44.95)		

The statistically significant variables were education level, presenting symptoms, subcortical structures and other territories involved, as in Table 4.

**Table 4:** Multivariate analysis of factors associated with outcomes among ischemic stroke patients at MNRH, KNRH and SFN MCA: Middle Cerebral Artery, M1: First Segment of the Middle Cerebral Artery, M2: Second Segment of the Middle Cerebral Artery, M3: Third Segment of the Middle Cerebral Artery, M4: Fourth Segment of the Middle Cerebral Artery, M5: Fifth Segment of the Middle Cerebral Artery, PCA: Posterior Cerebral Artery All variables with a p-value greater than 0.25 at bivariate analysis were excluded from the multivariable analysis. Adjusted odds ratios from a multivariable binary logistic regression model were used to determine the variables statistically associated with outcomes among ischaemic stroke patients. The LR chi² was used to determine 5% significance, and the overall significance of the model was < 0.001.

Variable (N=118)	uOR (95% CI)	P-Value	aOR (95% CI)	P-Value
Sex				
Male	1		1	
Female	1.77(0.67-4.67)	0.247	3.00 (0.57-15.70)	0.192
Education Level				
None	1		1	
Primary	073 (0.09-5.66)	0.764	0.34 (0.02-5.39)	0.44
Secondary	0.45 (0.84-2.40)	0.350	0.32 (0.03-3.44)	0.347
Tertiary	0.14 (0.03-0.71)	0.018	0.04 (0.01-0.58)	0.017
Presenting symptoms				
Facial paralysis	0.39 (0.14-1.12)	0.081	1.80 (0.19-17.55)	0.611
Dysarthria	0.39 (0.15-1.07)	0.068	0.17 (0.03-0.91)	0.039
MCA regions				
Caudate (U)	2.32 (0.72-7.4)	0.158	2.26 (0.37-13.95)	0.379
Internal Capsule (IC)	3.41 (1.99-5.86)	<0.001	0.99 (0.13-7.71)	0.995
Lentiform nucleus (IL)	4.52 (1.41-14.50)	0.011	4.06 (0.74-22.24)	0.107
Insular Ribbon (IR)	8.22 (2.26-29.90)	0.001	1.63 (0.15-18.41)	0.691
M1	8.29 (1.83-37.68)	0.006	0.83 (0.05-15.06)	0.900
M4	11.51 (2.53-52.34)		1.23 (0.03-44.34)	0.910
M3	19.79 (2.55-115.21)	0.004	13.32 (2.69-97.71)	0.047
M5	12.00 (2.64-54.57)	0.001	16.89 (1.04-102.88)	0.028
Other vascular territory				
PCA	5.18 (0.66-40.98)	0.119	13.98 (1.71-88.70)	0.051
Rehabilitation services				
Physiotherapy	0.23 (0.08-0.62)	0.004	0.30 (0.04-2.20)	0.238

There were poorer outcomes among patients with abnormal MCA involvement in these regions: Internal Capsule (IC) (p= 0.029), Lentiform nucleus (IL) (p= 0.007), Anterior MCA cortex (M1) (p= 0.002), Posterior cortex immediately rostral to M3 (M6) (p= 0.013), insular ribbon (IR), MCA cortex lateral to insular ribbon (M2), Posterior MCA (M3), anterior cortex immediately rostral to M1 (M4) and lateral cortex immediately rostral to M2 (M5), respectively (p< 0.001). Patients who had abnormal posterior MCA (M3) were about 13 times more likely to have poor outcomes compared to others (p= 0.047, aOR= 13.32). Abnormal lateral cortex immediately rostral to M2 (M5) were about 17 times more likely to have poor outcomes compared to others (p= 0.028, aOR= 16.89). Patients who had PCA were about 13 times more likely to have poor outcomes compared to others (p= 0.051, aOR= 13.98). Patients who had a tertiary education level were about 96% less likely to have poor outcomes compared to others (p= 0.017, aOR= 0.04), whereas patients who presented with signs and symptoms of dysarthria were 83% less likely to have poor outcomes compared to others (p= 0.039, aOR= 0.17).

## Discussion

Few studies have been reported in Sub-Saharan Africa regarding the usage and utilisation of the ASPECTS on the head NCCT. This study set out to determine the correlation between ASPECTS and the functional outcomes among ischaemic stroke patients at three selected centres. 

The median age was 60; most of the patients were less than 49 years old. Similarly, a mean age <65 years was found in other stroke studies [10-12]. These poor outcomes were also seen to be more prevalent among the 60 and above age group (n=58) as compared to ≤59 (n=40). Similarly, poor functional outcomes and higher morbidity and mortality were likely to occur in older patients [13,14]. Demographic trends revealed higher prevalence and poorer outcomes among older female patients, which may relate to age-related hormonal changes, comorbidities, and potentially better health-seeking behaviours among women. This could be attributed to better health-seeking behaviour and women generally having a higher life expectancy [10,11,13,15]. This study found a higher prevalence of ischaemic stroke among women, and this was similar to other study findings.

In bivariate analysis, poor outcomes were more common in women (n=58, 56.78%) as compared to men (n=40, 43.22%). Worse outcomes were also observed in women who had strokes [16,17], and death as an outcome was common among women aged 50-70 years [18]. Poor functional outcomes were also observed among elderly women with comorbidities [19].

Ischaemic infarcts were more prevalent in the left hemisphere, in keeping with previous studies [20-22]. There were poorer outcomes among patients who only had the left hemisphere affected (n=51, 56.7%) compared to those who had poorer outcomes with only the right hemisphere affected (n=39, 43.3%). Similarly, the left hemisphere was more commonly involved in ischaemic infarcts and was associated with an increased mortality [20]. Having both hemispheres involved was associated with poorer outcomes, as 66.7% of these had the worst outcome, death.

Over half of the patients had a GCS ≤9; n=65, 55.1%. Poor outcomes were associated with low GCS ≤13 at admission and lower ASPECTS as seen in bivariate analysis. Similarly, poorer functional outcomes were associated with a high National Institutes of Health Stroke Scale (NIHSS) score [23].

According to this study, being educated was protective against poorer functional outcomes, and this finding was found in another study that found higher education attained was associated with favourable outcomes and low education was associated with mortality and recurrent strokes [24,25] this was attributed to access to perceived information regarding stroke symptoms and seeking health services among those educated.

The majority of the participants received appropriate standard treatment as per the stroke hospital guidelines. This included non-thrombolytic therapy, antiplatelet therapy and lipid-lowering drugs. The patients received antihypertensive medication and oral hypoglycaemic or insulin therapy depending on the diagnosis or previous history. The choice of giving anticonvulsants or antibiotics was based on the clinical condition of the patient either developing seizures or respiratory problems or aspiration pneumonia and hospital-acquired infection. Early rehabilitation initiation among ischaemic stroke patients is associated with favourable outcomes [26,27]. Our study found only 31% were receiving rehabilitation services, and of these, a smaller percentage were only able to access them right away from the hospital admission. This is likely due to a poor referral system to the rehabilitation centres, financial constraints in accessing these services and a lack of knowledge regarding rehabilitation in ischaemic stroke patients.

A total of 18% of the patients had concurrent PCA involvement, and 3% had concurrent ACA involvement. Our study found that having an additional concurrent territory affected the PCA, which was associated with poorer outcomes. There are scant studies that have been done regarding MCA ischaemic strokes with concurrent territories involved; however, concurrent ACA involvement predicts mortality in severe MCA infarcts [28].

The average ASPECTS score in this study was 5.71, which is comparable to previous studies in similar settings [10], indicating that the patients are getting moderate to severe strokes. The majority of patients had a poor outcome with an mRS score ≥3, n=98 (83.1%). Despite more than half of the patients (n=68; 56.7%) being eligible for thrombolysis and thrombectomy, none received this therapy due to a lack of acute stroke reperfusion therapy protocols in these health facilities. The 30-day mortality from this study was 26.27%, similar to 26.8% found previously in the same setting [9]. Our 30-day mortality included in-hospital deaths; these deaths were attributed to aspiration pneumonia, possibly due to poor nursing care. A small proportion of these patients who died by day 30 had other comorbidities, including malignancy, chronic renal failure, and cardiac disease that may have contributed to their demise. In comparison to an inverse correlation of r = -0.39 at p = 0.04 between the ASPECTS and the mRS at three months [8], this study found an inverse correlation of -0.618 at p -< 0.001 at one month, indicating poorer outcomes. This could be attributed to stroke protocols that often utilise thrombolytic therapy and better rehabilitation services; with low ASPECTS, patients scored high mRS scores, translating to poor outcomes. Similarly, an inverse correlation between ASPECTS and the functional outcomes was observed [21], implying that the ASPECTS can reliably be used as a predictor for functional outcomes, and aggregated efforts can be made towards those with severe strokes with low ASPECTS during management and rehabilitation. At bivariate analysis, besides the overall total ASPECTS, all the individual MCA regions except the caudate nucleus were significant predictors of poor outcomes. However, in multivariate analysis, individual MCA regions were also found to be significantly associated with poor outcomes. The M4 caudate and the insula regions were found to be significantly related to poorer outcomes [29], whereas our study found M3 and M5 were statistically associated with poor outcomes. Thus, the specific MCA regions are also a predictor for functional outcomes in addition to the overall ASPECTS. Poor outcomes were also more frequent in left-hemisphere strokes and among patients with lower education levels, indicating possible socioeconomic and systemic health disparities impacting recovery and mortality rates. Although standard stroke treatments were administered according to hospital guidelines, the absence of acute reperfusion protocols and limited access to rehabilitation services likely contributed to higher 30-day mortality (26.27%). These findings stress the need to improve stroke care infrastructure, particularly in establishing thrombolysis protocols and expanding access to rehabilitation. Improved training, earlier stroke detection, and prompt intervention may help mitigate severe functional impairments and reduce mortality.

Limitations of the study

Some patients may have been misclassified as ischaemic stroke cases due to the unavailability of head-to-neck CTA and further imaging for comprehensive assessment. Delays in hospital presentation and limited rehabilitation accessibility further challenge the quality of stroke care in this setting. Inability to offer thrombolysis and thrombectomy to ischaemic stroke patients who present within the window. Some patients had other comorbidities that may have contributed to already having a pre-stroke disability. The variability in the rehabilitation services received across the three centres may have influenced the outcomes seen.

## Conclusions

This study highlights the value of the Alberta Stroke Program Early CT Score (ASPECTS) as a reliable predictor for functional outcomes in ischaemic stroke patients in a Sub-Saharan African setting. Our findings demonstrate a lower mean ASPECTS score (5.71) compared to studies in higher-resource settings indicating moderate to severe strokes among our patients. The majority of participants (83.1%) experienced poor functional outcomes (mRS ≥3) with an inverse correlation between ASPECTS and mRS similar to prior studies. This suggests that lower ASPECTS scores are associated with worse outcomes, particularly in the absence of reperfusion therapies like thrombolysis and thrombectomy, which are not available in these facilities. Poorer outcomes were significantly associated with lower ASPECTS scores and low GCS at admission, emphasising the role of these measures in early prognostication and potentially guiding resource allocation for stroke management and rehabilitation efforts. Specific MCA regions, notably M3 and M5, were also independently predictive of poor outcomes, underscoring the importance of regional ASPECTS scoring. 

## Appendices

**Table 5:** Data collection tool for patient demographics GCS: Glasglow Coma Scale, CT: Computed tomography

PARTICIPANT DEMOGRAPHICS		
1	Age	
2	Sex	
3	Nationality	
a)	Ugandan	
b)	Non-Ugandan	
4	Urbanity	
a)	Rural	
b)	Urban	
5	Education	
a)	Primary	
b)	Secondary	
c)	Tertiary	
6	GCS	
a)	3-8	
b)	9-13	
c)	14-15	
7	Comorbidities	
a)	Yes	
b)	No	
	If yes	
a)	Diabetes mellitus	
b)	Hypertension	
c)	others	
8	Subsequent imaging done	
a)	Yes	
b)	No	
9	Type of imaging done	
a)	CT scan	
b)	CT perfusion scan	
c)	MRI	
10	Presenting symptoms	
a)	Facial paralysis	
b)	Hemiparesis	
c)	Sensory loss of face or extremities	
d)	Aphasia	
e)	Dysarthria	
f)	Others	
11	Type of intervention received	
a)	Thrombolysis	
b)	Thrombectomy	
c)	Antihypertensive therapy	
d)	Antiplatelet therapy	
e)	Insulin therapy	
f)	Oral hypoglycemics	
g)	Lipid lowering therapy	
h)	Anticonvulsants	
i)	Others	

**Table 6:** Data collection tool for the ASPECTS MCA: Middle cerebral artery

FINDINGS ON NON-CONTRAST HEAD CT SCAN-ASPECTS		
Subcortical structures	Left	Right
Caudate (U)		
Internal capsule (IC)		
Lentiform nucleus (IL)		
MCA Cortex		
Insular ribbon (IR)		
Anterior MCA cortex (M1)		
MCA cortex lateral to the insular ribbon (M2)		
Posterior MCA (M3)		
Anterior cortex immediately rostral to M1 (M4)		
Lateral cortex immediately rostral to M2 (M5)		
Posterior cortex immediately rostral to M3 (M6)		
TOTAL		

**Table 7:** Data collection tool at follow-up During the follow-up phone calls made, functional outcomes were assessed using the mRS: Modified Rankin Scale. Participants and next of kin were also asked about any rehabilitation services received.

FUNCTIONAL OUTCOMES AND REHABILITATION	
mRS-description	Participant score
No symptoms	
No significant morbidity, carries out all usual activities despite some symptoms	
Slight disability; able to look after own affairs without assistance but unable to carry out all previous activities	
Moderate disability; requires some help but able to walk unassisted	
Moderately to severe disability; unable to attend to own bodily needs without assistance and unable to walk unassisted	
Severe disability; requires constant nursing care and attention, is bedridden, incontinent	
Dead	
Did the patient receive rehabilitation services?	
Physiotherapy	
Occupational therapy	
Speech and language therapy	

## References

[1] Katan M, Luft A (2018). Global burden of stroke. Semin Neurol.

[2] Feigin VL, Brainin M, Norrving B (2022). World Stroke Organization (WSO): Global stroke fact sheet 2022. Int J Stroke.

[3] Akbarzadeh MA, Sanaie S, Kuchaki Rafsanjani M (2021). Role of imaging in early diagnosis of acute ischemic stroke: a literature review. Egy Jr Neu Psy Neu.

[4] van Dam LF, van Walderveen MA, Kroft LJ (2020). Current imaging modalities for diagnosing cerebral vein thrombosis - a critical review. Thromb Res.

[5] Shafaat O, Sotoudeh H (2023). Stroke imaging. https://www.ncbi.nlm.nih.gov/books/NBK546635/.

[6] Caruso P, Ridolfi M, Lugnan C (2021). Multimodal CT pc-ASPECTS in infratentorial stroke: diagnostic and prognostic value. Neurol Sci.

[7] Schröder J, Thomalla G (2016). A critical review of Alberta stroke program early CT score for evaluation of acute stroke imaging. Front Neurol.

[8] Esmael A, Elsherief M, Razek AAKA (2021). Relationship of Alberta Stroke Program Early CT Score (ASPECTS) with the outcome of ischemic stroke and the neurocognitive stroke biomarkers. Egyp Jr Neu Psy Neu.

[9] Nakibuuka J, Sajatovic M, Nankabirwa J (2015). Early mortality and functional outcome after acute stroke in Uganda: prospective study with 30 day follow-up. Springerplus.

[10] Vincent M, Sereke SG, Nassanga R (2023). Correlation between clinical and brain computed tomography findings of stroke patients: A cross-sectional study. Health Sci Rep.

[11] Kwarisiima L, Mukisa R, Nakibuuka J (2014). Thirty-day stroke mortality and associated clinical and laboratory factors among adult stroke patients admitted at Mulago hospital (Uganda). Afr J Neurol Sci.

[12] Guzeldag S (2022). The effect of age and sex on ischemıc stroke: a sıngle-centred neuro-intensıve care unıt experıence. Acta Neurol Taiwan.

[13] Kamwesiga JT, von Kock LK, Eriksson GM, Guidetti SG (2018). The impact of stroke on people living in central Uganda: A descriptive study. Afr J Disabil.

[14] Viticchi G, Falsetti L, Plutino A (2020). Sex influence in ischemic stroke severity and outcome among metabolically unhealthy overweight patients. J Neurol Sci.

[15] Roy-O'Reilly M, McCullough LD (2018). Age and sex are critical factors in ischemic stroke pathology. Endocrinology.

[16] Dahl S, Hjalmarsson C, Andersson B (2020). Sex differences in risk factors, treatment, and prognosis in acute stroke. Womens Health (Lond).

[17] Yu AY, Austin PC, Rashid M (2023). Sex differences in intensity of care and outcomes after acute ischemic stroke across the age continuum. Neurology.

[18] Ohya Y, Matsuo R, Sato N (2023). Modification of the effects of age on clinical outcomes through management of lifestyle-related factors in patients with acute ischemic stroke. J Neurol Sci.

[19] Yousufuddin M, Young N (2019). Aging and ischemic stroke. Aging (Albany NY).

[20] Hedna VS, Bodhit AN, Ansari S (2013). Hemispheric differences in ischemic stroke: is left-hemisphere stroke more common?. J Clin Neurol.

[21] Hungerford JP, Hyer M, Turk AS (2017). Impact of ASPECT scores and infarct distribution on outcomes among patients undergoing thrombectomy for acute ischemic stroke with the ADAPT technique. J Neurointerv Surg.

[22] Portegies ML, Selwaness M, Hofman A (2015). Left-sided strokes are more often recognized than right-sided strokes: the Rotterdam study. Stroke.

[23] Esmael A, Elsherief M, Eltoukhy K (2021). Predictive value of the Alberta Stroke Program Early CT score (ASPECTS) in the outcome of the acute ischemic stroke and its correlation with stroke subtypes, NIHSS, and cognitive impairment. Stroke Res Treat.

[24] Che B, Shen S, Zhu Z (2020). Education level and long‐term mortality, recurrent stroke, and cardiovascular events in patients with ischemic stroke. J Am Heart Assoc.

[25] Braadt L, Meisinger C, Linseisen J (2022). Influence of educational status and migration background on the long-term health-related quality of life after stroke. Eur J Neurol.

[26] Wang F, Zhang S, Zhou F (2022). Early physical rehabilitation therapy between 24 and 48 h following acute ischemic stroke onset: a randomized controlled trial. Disabil Rehabil.

[27] Oyanagi K, Kitai T, Yoshimura Y (2021). Effect of early intensive rehabilitation on the clinical outcomes of patients with acute stroke. Geriatr Gerontol Int.

[28] Walcott BP, Miller JC, Kwon CS (2014). Outcomes in severe middle cerebral artery ischemic stroke. Neurocrit Care.

[29] Seyedsaadat SM, Neuhaus AA, Nicholson PJ (2021). Differential contribution of ASPECTS regions to clinical outcome after thrombectomy for acute ischemic stroke. AJNR Am J Neuroradiol.

